# Construction of an IFNAR1 knockout MDBK cell line using CRISPR/Cas9 and its effect on bovine virus replication

**DOI:** 10.3389/fimmu.2024.1404649

**Published:** 2024-07-19

**Authors:** Yuanchen Geng, Chuanwen Jiang, Hao Yang, Qing Xia, Xiaowen Xu, Kaihui Yang, Xinwei Yuan, Jianguo Chen, Yingyu Chen, Xi Chen, Lei Zhang, Changmin Hu, Aizhen Guo

**Affiliations:** ^1^ State Key Laboratory of Agricultural Microbiology, Hubei Hongshan Laboratory, College of Veterinary Medicine, Huazhong Agricultural University, Wuhan, China; ^2^ Hubei International Scientific and Technological Cooperation Base of Veterinary Epidemiology, The Cooperative Innovation Center for Sustainable Pig Production, Wuhan, China; ^3^ Key Laboratory of Development of Veterinary Diagnostic Products, Ministry of Agriculture and Rural Affair, Wuhan, China

**Keywords:** IFNAR1, BVDV, MDBK cell line, CRISPR/Cas9, SLC25A34, IL13RA2

## Abstract

The type I interferon (IFN) pathway is important for eukaryotic cells to resist viral infection, as well as an impediment to efficient virus replication. Therefore, this study aims to create an IFNAR1 knockout (KO) Madin-Darby bovine kidney (MDBK) cell line using CRISPR/Cas9 and investigate its application and potential mechanism in increasing viral replication of bovines. The IFNAR1 KO cells showed increased titers of bovine viral diarrhea virus (BVDV) (1.5 log10), with bovine enterovirus and bovine parainfluenza virus type 3 (0.5–0.8 log10). RNA-seq revealed reduced expression of the genes related IFN-I pathways including IFNAR1, STAT3, IRF9, and SOCS3 in IFNAR1 KO cells compared with WT cells. In WT cells, 306 differentially expressed genes (DEGs) were identified between BVDV-infected and -uninfected cells. Of these, 128 up- and 178 down-regulated genes were mainly associated with growth cycle and biosynthesis, respectively. In IFNAR1 KO cells, 286 DEGs were identified, with 82 up-regulated genes were associated with signaling pathways, and 204 down-regulated genes. Further, 92 DEGs were overlapped between WT and IFNAR1 KO cells including ESM1, IL13RA2, and SLC25A34. Unique DEGs in WT cells were related to inflammation and immune regulation, whereas those unique in IFNAR1 KO cells involved in cell cycle regulation through pathways such as MAPK. Knocking down SLC25A34 and IL13RA2 in IFNAR1 KO cells increased BVDV replication by 0.3 log10 and 0.4 log10, respectively. Additionally, we constructed an IFNAR1/IFNAR2 double-knockout MDBK cell line, which further increased BVDV viral titers compared with IFNAR1 KO cells (0.6 log10). Overall, the IFNAR1 KO MDBK cell line can support better replication of bovine viruses and therefore provides a valuable tool for bovine virus research on viral pathogenesis and host innate immune response.

## 1 Introduction

The interferon (IFN) system is a crucial component of the innate immune system and has significant impact on restricting viral replication and pathogenesis (1–3). Upon viral infection, the cells release IFNs, which attach to the IFN receptor on the cell membrane, triggering the internal signaling pathway and controlling the cellular development and function. This process liberates the cells from the viruses, thereby accomplishing the objective of fighting against viral infections. Notably, IFN-I signaling serves as a crucial defense mechanism in eukaryotic cells to combat viral invasion (2, 4). The receptor IFNAR1 and IFNAR2 collaborate in this pathway to trigger signal transducer and activator of transcription (STAT), leading to the activation of IFN-stimulated genes (ISGs) and the subsequent manifestation of antiviral effects within the cell (5–7). However, IFN itself does not play a direct role in killing or neutralizing viruses. Studies have indicated that mice lacking the IFNAR gene were more vulnerable to Zika virus infection, whereas chicken fibroblast cell lines lacking the IFNAR1 gene could greatly enhance the replication efficiency of duck Tembusu virus (2, 8, 9). However, to our knowledge, the effect of IFNAR1 knockout (KO) in bovine origin cells on bovine-derived virus replication has not been reported, and must be further studied.

Bovine viral pathogens play a critical role in calf diarrhea, such as bovine viral diarrhea virus (BVDV), bovine enterovirus (BEV) and bovine respiratory disease complex (BRD) such as bovine parainfluenza virus type 3 (BPIV-3). BVDV is an important pathogen for both calf diarrhea and BRD (10, 11). The isolation, identification, and replication of bovine viruses is important for clinical control, basic research, such as pathogenesis and immune response, and applied research, such as vaccine and diagnosis reagent development. IFNAR1 KO in bovine origin cells might be beneficial for highly efficient replication of bovine viruses. The CRISPR/Cas9 system has become the most commonly used gene editing technology at present (12–14), it would be an ideal tool to develop bovine IFNAR1 KO cells.

This study aimed to create an IFNAR1 KO Madin-Darby bovine kidney (MDBK) cell line (designated KO MDBK) using CRISPR/Cas9 and grow BVDV, BPIV-3, and BEV significantly better in the IFNAR1 KO MDBK cell line. In addition, RNA-seq analysis was used to identify differentially expressed genes (DEGs) and mechanisms that contribute to increase in viral replication.

## 2 Materials and methods

### 2.1 Cells and viruses

MDBK and 293T cells were purchased from China General Microbiological Culture Collection Center. The cells were cultured in a humidified incubator at 37°C with 5% CO_2_ using Dulbecco’s modified Eagle’s medium (DMEM) supplemented with 10% fetal bovine serum (FBS) and 1% penicillin-streptomycin.

BEV (GenBank accession number:LC150008), BVDV type 1 AV69 VEDEVAC strain (GenBank accession number: KC695814), and BPIV-3 (GenBank accession number: JQ063064.1) were stored in our laboratory.

### 2.2 Establishment of IFNAR1 KO MDBK cell line

CRISPR/cas9 was used to establish IFNAR1 KO cells. Four sgRNAs were designed according to the sequence of IFNAR1 (Gene ID:282257) in NCBI with the E-CRISP tool, and four pairs of primers were designed for each of the four sgRNAs for lentiviral vector plasmid (lenti-IFNAR1-sgRNA-pKLV2) construction.

To generate lentivirus, the sgRNA plasmid (Addgene) was cotransfected with 12 μg, along with 4 μg of pMD2.G plasmid (Addgene) and 8 μg of psPAX2 (Addgene) plasmid, into a 100 mm dish. The transfection was performed using JetPRIME (PolyPlus, Strasbourg, France) following the instructions provided by the manufacturer. After 60 h of transfection, the supernatant of 293T cells was passed through a 0.45 μm membrane with low protein binding (Millipore, Billerica, MA, USA), and centrifuged at 30,000 rpm and 4°C for 2.5 h. The virus pellets were reconstituted in phosphate-buffered saline (PBS) with pH 7.4, divided into smaller portions, and preserved at -80°C. Lentiviruses were introduced to MDBK-Cas9 cells in the presence of 8 μg/mL polybrene (Sigma Aldrich, St. Louis, MO, USA). After 24 h of transfection, the viruses were eliminated and substituted with new media. Following a 2 days period of recuperation, cells were provided with 2 µg/mL puromycin (Sigma Aldrich, St. Louis, MO, USA) in the medium for 7 days until selection was accomplished.

The genomic DNA of individual clones was sequenced after screening and picking single colonies. Each clone was transferred to a new 6-well plate and cultured until confluence. The genotypes of the colonies were examined through the extraction and sequencing of genomic DNA using TIANamp Genomic DNA Kit (TIANGEN, Beijing, China). The primers used for plasmid construction and sequencing are listed in **Table 1**.

**Table 1:** The primers of IFNAR1 used for plasmid construction.

sgRNA	sgRNA sequences	Primer sequences for plasmid construction (5’-3’)
sgRNA1	TTAGGTATAGCGTCGTTATC	F: caccgTTAGGTATAGCGTCGTTATC
		R: aaacGATAACGACGCTATACCTAAc
sgRNA2	GTCCGTGTACGAGCATCTAA	F: caccgGTCCGTGTACGAGCATCTAA
		R: aaacTTAGATGCTCGTACACGGACc
sgRNA3	GTCATCAGCGTGAAATCCGT	F: caccgACGGATTTCACGCTGATGAC
		R: aaacGTCATCAGCGTGAAATCCGTc
sgRNA4	GGGCGCGACGACCCTGATGC	F: caccgGCATCAGGGTCGTCGCGCCC
		R: aaacGGGCGCGACGACCCTGATGCc

### 2.3 Assay for detecting T7 endonuclease I cleavage

Mutation rate was determined with the T7 endonuclease I (T7EI) cleavage detection assay. First, genomic DNA was extracted from mutated polyclonal cells using TIANamp Genomic DNA Kit (TIANGEN, Beijing, China). PCR was performed with TaKaRa LA Taq (TaKaRa, Tokyo, Japan) to amplify the gene fragments of off-target sites using 35 cycles. The PCR products were purified and digested with T7EI (NEB, Beverly, MA, USA) for 15 min and separated with 2% agarose gel electrophoresis. Gel-Red was used to stain agarose gels, and the DNA signal in the gel was measured using Bio-Rad’s Image Lab software through densitometry. **Supplementary Table 1** contains a comprehensive list of all primers.

### 2.4 CCK-8 assay

The growth of wild-type (WT) and IFNAR1 KO cells were evaluated using Cell Counting Kit-8 (CCK-8) assay. The cells were placed one by one in 96-well plates (4 × 10^3 cells/well) and mixed with 10 µL of CCK-8 reagent (Vazyme, Nanjing, China). Signals were measured at an absorption wavelength of 450 nm at 12, 24, 36, 48, 60, and 72 h post CCK-8 treatment.

### 2.5 EdU cell proliferation assay

To evaluate cell proliferation, WT and IFNAR1 KO cells were seeded into 6-well plates. The cells were cultured in DMEM medium supplemented with 10% FBS and 1% penicillin-streptomycin at 37°C with a 5% CO_2_ atmosphere. At 24 h after incubation, EdU cell proliferation assays were performed using BeyoClick EdU Cell Proliferation Kit with Alexa Fluor 555 (Beyotime, Shanghai, China), according to the manufacturer’s instructions. DAPI (Beyotime, Shanghai, China) was used to stain nuclei for 10 min at room temperature in the dark. Fluorescence microscopy was used to observe the cells that had been stained. ImageJ software was used to calculate the ratio of EdU-positive cells. Imaging was performed for three wells, with one random field captured from each well.

### 2.6 RNA extraction and RT-qPCR analysis

Cells were subjected to RNA extraction using TransZol Up (TransGen, Beijing, China) and viral RNAs were extracted from cell suspensions using Viral RNA Extraction Kit (TaKaRa, Tokyo, Japan) according to the manufacturer’s instructions. To evaluate the quantity and quality of RNA, a NanoDrop 2000 spectrophotometer (Thermo Fisher Scientific, Waltham, MA, USA) was used. PrimeScript RT reagent Kit with gDNA Eraser (TaKaRa, Tokyo, Japan) was used to synthesize cDNAs. The cDNAs were used as templates for relative quantitative real-time qPCR. The qPCR reactions were performed using Real Universal SYBR Green Premix (Vazyme, Nanjing, China) according to the instructions provided by the manufacturer. CFX96 Real-Time PCR System (Bio-Rad, Hercules, CA, USA) was used to monitor the results, with a programmed sequence of one cycle lasting 10 min at 95°C, followed by 40 cycles of 10 s at 95°C and 30 s at 60°C. The 2^−ΔΔCt^ method was used to calculate relative expression. The GAPDH gene served as a control for normalization. Each experiment was conducted independently three times. Gene specific primers for qPCR are shown in **Table 2**.

**Table 2:** The gene specific primers for qPCR.

Genes	Forward primers (5’-3’)	Reverse primers (5’-3’)
BEV(5’UTR)	TTCACGGCCCCGGAGGAAAA	TCGCACATCCCCCGTGGTAA
BPIV-3(NP)	CACGTGATGCAGAATCGCAG	GATTCGGGCTGCTCTGCTT
BVDV(5’UTR)	TAGTCGTCAGTGGTTCGACGC	CCTCTGCAGCACCCTATCAG
GAPDH	ACCCAGAAGACTGTGGATGG	CAACAGACACGTTGGGAGTG
IFNAR1	TTCCTCCAGTCATCAGCGTG	GCGGGTAGAGCTGATTCACA
IFN-β	CTGCAGCCAATCCAGAAGGA	CCTTGGACTCCAGGTACTGC
Mx	GTTGGTCTACTGCCAGGACC	TCATCTGTGGAGGGCTCTGA
IFIT5	ATCAAGAAGGCCACTCGCAA	CCAGGTCCGTGTAGGCAAAT
IRF1	AAAAGGAGCCGGATCCCAAG	TCGGCTGGACTTGGACTTTC
ISG15	TGAAGCAGACTGTGGCTGAG	CTCATCATCCATGGGCCTCC
SLC25A34	GCCCTGCTTACCTGGTCAAA	AGGCTCTCGTGATGGTGTTG
SLC25A20	TATGACCCCTGGAGAGCGAA	CCCCTTGTAGATCCCTCGGA
SPRY4	CTCTGACCAGCGCCTCTTAG	TTGCACTTACACTTCCCGCA
TGM3	CCCATTGGAAGGTACACGCT	TATCAGCTTGCAACCAGGGG
ESM1	CTCGGAGAAACCTGCTACCG	TTGCATTCCATCCCGAAGGT
IL13RA2	ACCTTTGCCACCAGACTACC	TGGTAGTCACCCAGGCAGTA
SPP1	GCCACAGAGGAGGACTTCAC	TTATCCTTGGCCTTTGGCGT
TMEM252	TGGGGGCCTTCTTCATTTCC	GATCACAAACCCCAGAGGCA
BATF3	AGGCTGACAAACTCCACGAG	GCAAGGGCACAAAGTTCAGG
IL18RAP	GCCCAGAACTCCATTGGGAA	CGTCTTGTCCTTGCTCCAGT
NMUR2	ACCTCTTCAGCTTGGCTGTC	AACAGGAAGGGGTAGTTGCG
VGF	AGCCTCATTGAGCTGTCCAC	TTCTTCTTCCGCTTCCGCTT

### 2.7 TCID_50_ assay

Virus titers were determined using the TCID_50_ assay. The viruses were 10-fold serially diluted with DMEM from 10^−1 to 10^−8 in separate 1.5 mL Eppendorf tubes. A suspension of 5 × 10^5 MDBK cells/mL was placed in a 96-well plate with 100 µL of DMEM supplemented with 2% FBS and 1% penicillin-streptomycin per well. The cells were cultured in an incubator at 37°C for 2 h. Then 100 µL of diluted virus samples were added to each well and each dilution was repeated in eight wells of one column. Subsequently, the infected cells were incubated at 37°C for 48–96 h. The negative control column was set up with only cells in eight wells. The cytopathogenic effect (CPE) positive control column was set up with cells and 100 TCID_50_ of virus in eight wells. The presence of CPE in all wells was observed under a light microscope, and 50% tissue culture infectious dose (TCID_50_) was calculated using the Spearman-Karber method (15).

### 2.8 Rabbit and mouse antisera against bovine viruses

The rabbit antiserum against recombinant rVP2 of BEV was prepared, as described (16). This animal experiment protocol (HZAURAB-2022-0006) was approved by the Ethics Committee for Animal Experimentation of Huazhong Agricultural University and was conducted in strict accordance with the Guidelines for the Care and Use of Laboratory Animals of Hubei Province, China. The prokaryotic expression vector pET-28a is maintained in our laboratory. The full-length sequence of BEV-VP2 was amplified using the primers BEV-VP2–1F/1R (**Supplementary Table 2**) from BEV-F strain Xinjiang15, isolated, identified, and stored by our laboratory. Its VP2 sequence has 100% similarity with the public sequence (GenBank accession number: LC150008). The primers contained *Bam*HI and *Xho*I sites to allow the amplified VP2 sequence to be cloned into the pET-28a expression vector. The constructed recombinant plasmid, designated pET-BEV-VP2, was confirmed to be correct and transformed into competent BL21. The expression of recombinant VP2 was induced by 0.8 mM isopropyl-β-D-thiogalactoside (IPTG) (Sigma Aldrich, St. Louis, MO, USA) for 6 h, and the bacterial culture was harvested by centrifugation at 8,000 rpm for 15 min at 4°C. Recombinant VP2 expression was checked with 10% sodium dodecyl sulfate-polyacrylamide gel electrophoresis (SDS-PAGE) for (**Supplementary Figure 2A**). rVP2 with His tag was purified by nickel-affinity chromatography, as described (16), and confirmed with western blotting using a mouse anti-His tag mAb antibodies (Abbkine, Wuhan, China) (**Supplementary Figure 2B**).

Three male New Zealand white rabbits (1.5–2 kg) were used to develop polyclonal antibodies to rVP2. The rabbits were given a hypodermic injection of rVP2 protein (600 µg), mixed with Complete Freund’s Adjuvant (Sigma-Aldrich; St. Louis, MO, USA) in a 1:1 ratio. At 3 weeks after priming, the rabbits were boosted with an equal amount of VP2 (600 µg) but with incomplete Freund’s adjuvant (1:1). The third booster was given after 2 weeks. After 1 week, the rabbits were bled at the marginal vein of the ear, and sera were tested with homemade iELISA by using rVP2 as the coating antigen. When the antibody titer reached 1 × 10^4, the rabbits were euthanized and bled to obtain the antiserum.

The mouse monoclonal antibodies against BVDV (titer: 1.024 × 10^6) and BPIV-3 (titer: 8.192 × 10^5) were developed and kindly provided by our previous labmates Mengya Zhang and Yiting Long, respectively.

### 2.9 Western blot analysis

The cells were harvested with centrifugation at 10,000 rpm for 20 min at 4°C at different time points after infection with the virus. The cell pellet was lysed with RIPA lysis buffer containing 1% protease inhibitor cocktail. The BCA protein assay kit was used to measure the concentration of total proteins. Then protein samples were separated using 10% SDS-PAGE and transferred onto a polyvinylidene difluoride (PVDF) membrane. Next, the membrane was blocked using 5% skim milk powder at room temperature for 3 h; incubated overnight at 4°C with homemade mouse anti-BVDV and -BPIV-3 primary antibodies (1:1,000) and rabbit anti-BEV primary antibodies (1:500); washed three times with TBST (Biosharp, Guangzhou, China); and incubated with commercial HRP-conjugated goat IgG against mouse IgG for BVDV and BPIV-3 and rabbit IgG for BEV as the secondary antibodies (Abbkine, Wuhan, China) for 1 h. finally, the membranes were subjected to chemiluminescence detection.

### 2.10 Transcriptome sequencing analysis

Transcriptomic analysis was conducted by Majorbio Technologies Co., Ltd., (Shanghai, China). RNA-Seq was used to detect DEGs in BVDV-infected and -uninfected WT and IFNAR1 KO cells to identify the pathways of innate immunity. BVDV was used to infect the cells at a multiplicity of infection (MOI) of 0.1 for 24 h. Then, the cells were washed three times with PBS, and total RNA was extracted using TRIzol Reagent. The Illumina HiSeq sequencing technology platform was used to generate sequencing data by constructing a transcriptome library from the samples. Based on the quantitative expression results, the DEGs between the two groups were obtained using DESeq2 software, with a screening threshold of |log2FC| >=1 & *p* adjust < 0.05.

### 2.11 Transfection of siRNAs

The siRNAs were synthesized by TsingKe (Beijing, China). Cells lacking IFNAR1 were plated onto 6-well plates and transfected with 110 pmol siRNA using Lipofectamine 2,000 (Invitrogen, Carlsbad, CA, USA), as per the instructions provided by the manufacturer. The cells were approximately 60% confluent. Cells transfected with a siRNA-NC served as negative controls. Cells were infected with BVDV at 0.1 MOI, 12 h after transfection. The infected cells were incubated for 12, 24, 36, and 48 h postinfection (hpi). Subsequently, the treated cells were used for additional functional analysis. The siRNA sequences of the gene are shown in **Table 3**.

**Table 3:** The siRNA sequences of the genes.

Genes	Sense sequences (5’-3’)	Antisense sequences (5’-3’)
SLC25A34	GCCCUGCUUACCUGGU CAA	UUGACCAGGUAAGCAG GGC
IL13RA2	GGAUAGCAAUUACAG GUUA	UAACCUGUAAUUGCUA UCC

### 2.12 Establishment of IFNAR1 complement cell lines

The amino acid near NGG in the IFNAR1 CDs region sgRNA4 was mutated to synonymous amino acids to obtain the IFNAR1 gene complement sequence. The *Eco*RI sequence was added before the start codon of the complement sequence. The FLAG tag was added before the stop codon. The *Bam*HI sequence was added after the stop codon. The PCR products were homologously recombined to pLVX to generate the pLVX-IFNAR1. Lentiviral packaging and infection of IFNAR1 KO cells were performed as described in section 2.2. Finally, the IFNAR1 complement cell line was obtained by neomycin drug screening.

### 2.13 Establishment of IFNAR1 and IFNAR2 double-knockout cell lines

According to the IFNAR2 (Gene ID: 282258) sequence in NCBI, three sgRNAs were designed using E-CRISP, and the knockout plasmid pKLV2-IFNAR2-sgRNA was constructed for the three sgRNAs. The steps are shown in 2.2. IFNAR2 sgRNA sequences and synthetic primers are shown in **Supplementary Table 3**

### 2.14 TA clone verification

First, genomic DNA was extracted from mutated polyclonal cells using TIANamp Genomic DNA Kit (TIANGEN, Beijing, China). PCR was performed with TaKaRa LA Taq (TaKaRa, Tokyo, Japan) to amplify the gene fragments of off-target sites using 35 cycles. The PCR products were purified and ligated into the pMD19T vector (TaKaRa, Tokyo, Japan) and subsequently transformed into *DH5α* competent cells (TaKaRa, Tokyo, Japan). The transformed cells were then plated on LB agar plates containing ampicillin and incubated at 37°C overnight. Individual colonies were selected and submitted to Tsingke company for sequencing.

### 2.15 Statistical analysis

Data are presented as means ± standard errors of the mean (SEMs) of triplicates. GraphPad Prism software (Version 9.5.0, Boston, MA, USA) was used for statistical analysis. Statistical significance was determined using Student’s *t*-test for a single comparison and analysis of variance (ANOVA) for more than one comparison. Differences in *p* values of <0.05 (*), <0.01 (**), <0.001 (***) and <0.0001 (****) were considered statistically significant, whereas a *p* value of >0.05 was not considered statistically significant.

## 3 Results

### 3.1 Construction of IFNAR1 KO cell line using CRISPR/Cas9

**Figure 1:**
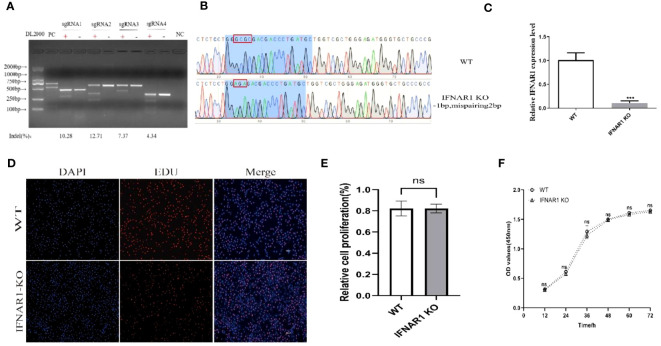
Construction of IFNAR1 KO MDBK cell line. **(A)** T7 endonuclease I cleavage detection to verified sgRNA mutation efficiency. **(B)** Snapgene software was used to confirm the sequencing peaks of PCR products from WT and IFNAR1 KO MDBK cells. **(C)** Relative quantitative PCR was used to detect transcript levels in the IFNAR1 KO cells. **(D)** EdU cell proliferation assay was applied to detect proliferation of IFNAR1 KO cells. **(E)** The analysis results of EdU cell proliferation assay data. **(F)** The growth rate of the IFNAR1 KO cells were detected by cell growth curves using the CCK-8 assay. For **(C, E, F)**, ***p < 0.001; ns, non-significant.

Four knockout plasmid pKLV2-IFNAR1-sgRNAs were created by using four distinct sgRNA sequences targeting various locations within the IFNAR1 exon. Each of the four plasmids was separately transfected into MDBK-Cas9 cells, and IFNAR1 KO cells were screened with puromycin. The T7EI zymography assay confirmed that all four sgRNAs could generate polyclonal IFNAR1 KO cells (**Figure 1A**). sgRNA2 KO cells with the highest indel rate and sgRNA4 KO cells with the lowest indel rate were selected for phenotypic validation. KO cells with sgRNA4 were more effective in increasing viral titers and therefore, selected for subsequent experiments (**Supplementary Figures 1A, B**). To ensure that the observed effects in this study could be directly attributed to the knockout of the IFNAR1 gene, we utilized lentiviral packaging of the lenti-pKLV2 empty plasmid to transfect MDBK-Cas9 cells as a negative control. Our findings indicate that there was no significant difference in the impact on viral replication between the vector-transfected and WT cells (**Supplementary Figures 1C, D**).

The IFNAR1-sgRNA4 KO cells were screened using the restriction dilution method to obtain monoclonal cell lines with IFNAR1 KO. DNA sequencing revealed that the picked monoclonal cell line contained pure mutations in the IFNAR1 gene (**Figure 1B**). RNA from IFNAR1 KO cells was extracted and analyzed using relative quantitative PCR, indicating a significant decrease in IFNAR1 KO transcripts compared with WT MDBK cells (**Figure 1C**). The proliferation of IFNAR1 KO MDBK cells was unaffected by IFNAR1 knockout, as indicated by the results of the EdU cell proliferation assay (**Figures 1D, E**). Growth rate was evaluated by creating cell growth curves using the CCK-8 assay and measuring absorbance at 450 nm. The findings indicated no notable disparities in growth rates between WT and IFNAR1 KO MDBK cells (**Figure 1F**). The results validated the effective establishment of a consistently passaged IFNAR1 KO MDBK cell line with IFNAR1 knockout, which was used for subsequent experiments.

### 3.2 Knockout of IFNAR1 significantly increased viral titers

**Figure 2:**
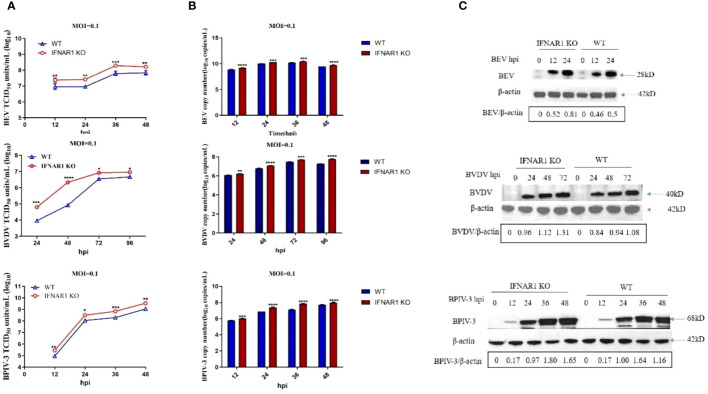
Knockout of IFNAR1 gene significantly increased viral titers of BEV, BVDV and BPIV-3. **(A)** WT and IFNAR1 KO MDBK cells were infected with BEV, BVDV and BPIV-3 at 0.1 MOI, respectively, and viral fluids were harvested at different time points. Viral titers were determined by TCID_50_ (Log10 TCID_50_/mL). **(B)** Viral RNA levels of BEV, BVDV and BPIV-3 were determined and presented by RT-qPCR. For **(A, B)**, *p < 0.05; **p < 0.01; ***p < 0.001; ****p < 0.0001. **(C)** Total cell lysates were prepared in parallel. Western blot assay was used to detect the replication of BEV, BVDV and BPIV-3, and β-actin was used as an internal reference.

To evaluate the impact of IFNAR1 knockout on viral replication, WT and IFNAR1 KO MDBK cells were infected with BEV, BVDV, and BPIV-3 at 0.1 MOI each. Viruses were harvested at various time points postinfection, depending on replication cycle. Viral titers in the medium were determined using TCID_50_. Levels of viral RNA and proteins were determined using RT-qPCR and western blotting assay, respectively. At 36 hpi, the titer of BEV in IFNAR1 KO was 0.6 log10 higher than that in WT. When infected with BPIV-3, viral titer of IFNAR1 KO at 12–48 hpi, was 0.5–0.8 log10 higher than that of WT-amplified virus. During the initial phases of BVDV replication, specifically at 24 and 48 hpi, the absence of IFNAR1 greatly amplified BVDV load, exhibiting a 1–1.5 log10 increase compared with WT (**Figure 2A**). The increase in viral titers corresponded to an accelerated rate of viral replication. Viral RNA collected at various time intervals was extracted and reverse transcribed to cDNA. Viral growth was then measured using RT-qPCR and viral growth curves were obtained. The results confirmed that IFNAR1 KO significantly increased viral RNA levels to varying degrees throughout the replication cycles of BEV, BVDV, and BPIV-3 compared with WT cells, especially when infected with BPIV-3 for 36 hpi (**Figure 2B**).

The western blotting assay demonstrated that the rabbit antiserum against BEV reacted with both rVP2 and BEV, with optimal dilution at 1:500 (**Supplementary Figure 2C**), whereas the homemade mouse antiserum against BVDV and BPIV-3 could react with the corresponding viruses, with optimal dilution at 1:1,000 (**Supplementary Figures 2D, E**). These antisera were used to detect viral replication in WT or IFNAR1 KO MDBK cells at various time points postinfection depending on viral replication properties (0, 12, and 24 hpi for BEV; 0, 24, 48, and 72 hpi for BVDV; and 0, 12, 24, 36, and 48 hpi for BPIV-3). Overall, the change in viral protein level followed the trend of change in viral RNA (**Figure 2C**).

The reintroduction of IFNAR1 in IFNAR1 KO cells in complementary cells decreased the replication of BEV, BVDV, and BPIV-3 to levels comparable to those in WT cells (**Supplementary Figure 3**). These findings indicate that the absence of IFNAR1 greatly increased the replication of BEV, BVDV, and BPIV-3.

### 3.3 IFNAR1 KO cells significantly reduce induction of ISGs after BEV, BPIV-3, and BVDV infection

**Figure 3:**
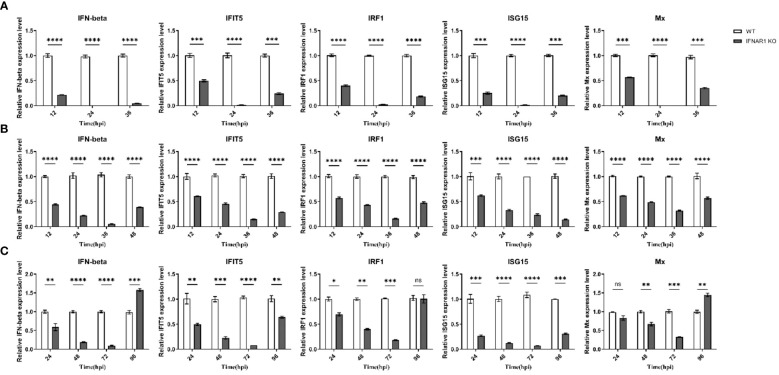
Induction of ISGs was significantly reduced by IFNAR1 KO cells infected with BEV, BPIV-3 and BVDV, respectively. RT-qPCR was performed to detect the expression levels of IFN-β, IFIT5, IRF1, ISG15, and Mx mRNA in BEV **(A)**, BPIV-3 **(B)**, and BVDV **(C)** infected cells. *p < 0.05; **p < 0.01; ***p < 0.001; ****p < 0.0001; ns, non-significant.

Samples were obtained from virus-infected WT and IFNAR1 KO MDBK cells at various time points. Total RNA was extracted from the samples and analyzed using RT-qPCR to confirm the impact of IFNAR1 knockout on the activation of type I IFN pathway during viral infection. Cytopathic abnormalities disappeared 36 h after BEV infection and were only detected at 12, 24, and 36 hpi. At 12 h after BEV infection of WT cells, IFN-β, one of type I IFN and four frequently observed ISGs (IFIT5, IRF1, ISG15, and Mx) exhibited substantial induction. Conversely, in IFNAR1 KO cells, these five genes were notably suppressed, with the trend being most prominent at 24 h after BEV infection (**Figure 3A**). After BPIV-3 infection, these five genes in IFNAR1 KO cells were inhibited to varying degrees during viral replication, most significantly at 36 h after BPIV-3 infection (**Figure 3B**). Similarly, after BVDV infection, these five genes were greatly induced in WT cells, whereas the level of induction was greatly inhibited at 72 h in IFNAR1 KO cells (**Figure 3C**). Taken together, these findings suggest that the absence of IFNAR1 greatly inhibited the activation of the type I IFN pathway, providing further evidence that IFNAR1 KO compromises IFN-I activity. At the late stage of viral replication, the induction level of ISGs in IFNAR1 KO cells was restored, most prominently in BVDV infection. This restoration may be attributed to the activation of alternative signaling mechanisms in response to viral invasion.

### 3.4 DEGs between WT and IFNAR1 KO MDBK cells infected and uninfected with BVDV

**Figure 4:**
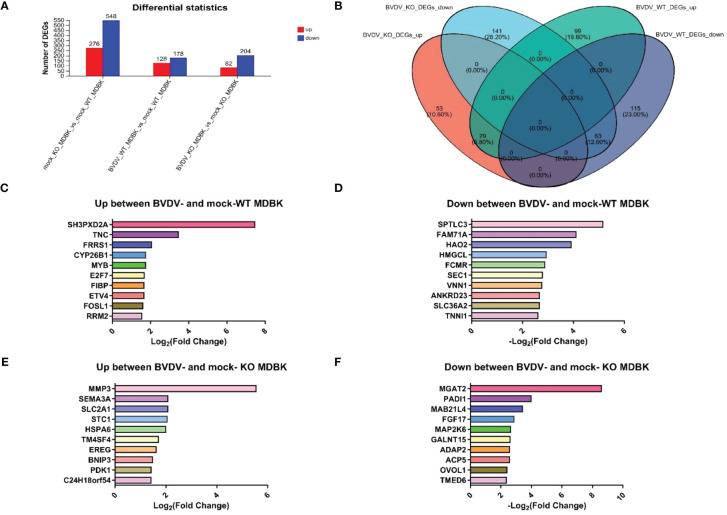
Transcriptomic analysis of DEGs in BVDV-infected and -uninfected WT and IFNAR1 KO MDBK groups. **(A)** The number of up-regulated DEGs in BVDV-infected cells at 24 hpi is shown in red, while down-regulated DEGs in blue. **(B)** Wayne diagram analysis on the DEGs between the WT and IFNAR1 KO MDBK groups with and without BVDV infection. **(C)** The top 10 up-regulated DEGs in the infected and uninfected WT MDBK groups. **(D)** The top 10 down-regulated DEGs in the infected and uninfected WT MDBK groups. **(E)** The top 10 up-regulated DEGs in the infected and uninfected IFNAR1 KO MDBK groups. **(F)** The 10 down-regulated DEGs in the infected and uninfected IFNAR1 KO MDBK groups.

WT and IFNAR1 KO MDBK cells infected and uninfected with BVDV were collected. Total RNA was extracted and analyzed by transcriptome sequencing. Sequencing results identified 824 DEGs in IFNAR1 KO cells compared with WT cells, including 276 up- and 548 down-regulated genes, of which the IFN pathway-related genes IFNAR1, STAT3, IRF9, and SOCS3 were significantly downregulated. These findings confirm that IFNAR1 knockout impaired the IFN signaling pathway. In WT cells, 306 DEGs were identified, consisting of 128 up- and 178 down-regulated genes between the infected and uninfected groups. In IFNAR1 KO cells, 286 DEGs were identified, comprising 82 up- and 204 down-regulated genes between the infected and uninfected groups (**Figure 4A**).

Wayne diagram analysis of DEGs between WT and IFNAR1 KO MDBK cells with and without BVDV infection showed that 92 identical DEGs overlapped, 29 were up-regulated, such as ESM1, IL13RA2, TMEM252, IL18RAP, and SLC25A34, and 63 were down-regulated, such as PADI2, PBLD, C1R, TTR, and GM2A (**Figure 4B**). In addition, 99 up-regulated DEGs, such as SH3PXD2A, TNC, FRRS1, CYP26B1, and MYB (**Figure 4C**), and 115 down-regulated DEGs, such as SPTLC3, FAM71NA, HAO2, HMGCL, and FCMR, were unique to the WT group (**Figure 4D**), whereas 53 up-regulated DEGs, such as MMP3, SEMA3A, SLC2A1, STC1, and HSPA6 (**Figure 4E**), and 141 down-regulated DEGs, such as MGAT2, PADI1, MAB21L4, FGF17, and MAP2K6, were unique to the IFNAR1 KO MDBK group (**Figure 4F**).

### 3.5 KEGG and GO analysis of DEGs in BVDV-infected and -uninfected WT and IFNAR1 KO MDBK cells

**Figure 5:**
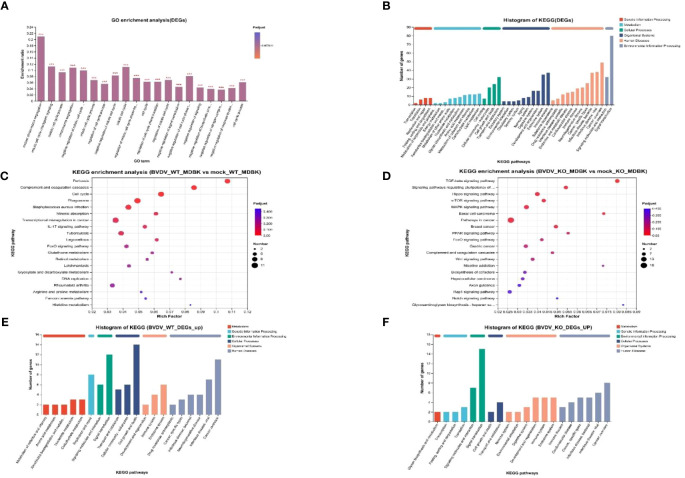
Biological significance of DEGs in BVDV-infected and -uninfected WT and IFNAR1 KO MDBK groups. **(A)** GO enrichment analysis of DEGs. The x-axis indicates the GO terms, while the y-axis indicates the rich factors of GO and column color gradient indicates the significance of enrichment. *p < 0.05; **p < 0.01; ***p < 0.001; **(B)** KEGG analysis of DEGs. **(C, D)** represent KEGG enrichment analysis of DEGs in BVDV-infected and -uninfected WT and IFNAR1 KO MDBK groups, respectively. In **(C, D)**, the x-axis indicates the rich factors, while the y-axis indicates the KEGG pathway terms. **(E, F)** indicate KEGG annotation analysis of upregulated DEGs in the BVDV-infected and -uninfected WT and IFNAR1 KO MDBK groups, respectively. In **(B, E, F)**, the x-axis indicates the different KEGG pathways, while the y-axis indicates the number of DEGs.

To study DEGs in WT and IFNAR1 KO MDBK cells after BVDV infection, GO enrichment analysis was performed. The DEGs were involved in molecular functions and biological processes, including signal transduction, biosynthesis process, nitrogen compound metabolism process, and stimulus response of negative regulation (**Figure 5A**). According to the KEGG annotation analysis, the DEGs primarily participate in signaling, interactions of signaling molecules, viral infections, immune system, and endocrine system (**Figure 5B**). KEGG enrichment analysis showed that BVDV infection of WT cells mainly activated regulatory pathways, such as cell cycle, phagolysosome, complement, and coagulation cascade response (**Figure 5C**). By contrast, BVDV infection of IFNAR1 KO cells predominantly activated signaling pathways such as MAPK, Hippo, TGF-β, mTOR, etc. (**Figure 5D**). KEGG annotation analysis of upregulated DEGs in the infected group showed that in the WT MDBK group, the DEGs were mainly involved in the regulatory pathways of cell growth and death, signaling, replication, and repair (**Figure 5E**), whereas DEGs in the IFNAR1 KO MDBK group, were mainly involved in signaling (**Figure 5F**). The signaling pathways in which DEGs were significantly enriched are shown. Important metabolic and signaling pathways involved in DEGs include the MAPK, PI3K-Akt, Hippo, and TGF-β signaling pathways, which are mainly regulated in the antiviral innate immune response triggered by BVDV infection (**Supplementary Figure 4A**). Up-regulated DEGs from the infected group were separately enriched and analyzed. DEGs in the WT MDBK group were mainly involved in cell cycle regulation. DEGs in the IFNAR1 KO MDBK group were mainly involved in the MAPK signaling pathway (**Supplementary Figure 4B**). The DEGs down-regulated in IFNAR1 KO MDBK cells infected with BVDV were mainly involved in cancer, Hippo, TGF-β, and mTOR pathways (**Supplementary Figure 4C**). These results suggest that DEGs in BVDV-infected WT cells are mainly involved in inflammation and immunomodulation, whereas DEGs in IFNAR1 KO cells are functionally involved in regulating the cell cycle through signaling, as well as regulation of cell proliferation, differentiation, and apoptosis. This indicates that cells depend on alternative mechanisms to defend against viral invasion following IFNAR1 knockout. Consistently, the results of clustering analysis in cell growth and death and disease immune system pathways confirmed this conclusion (**Supplementary Figures 4D, E**).

### 3.6 Validation of transcriptomic data using RT-qPCR

**Figure 6:**
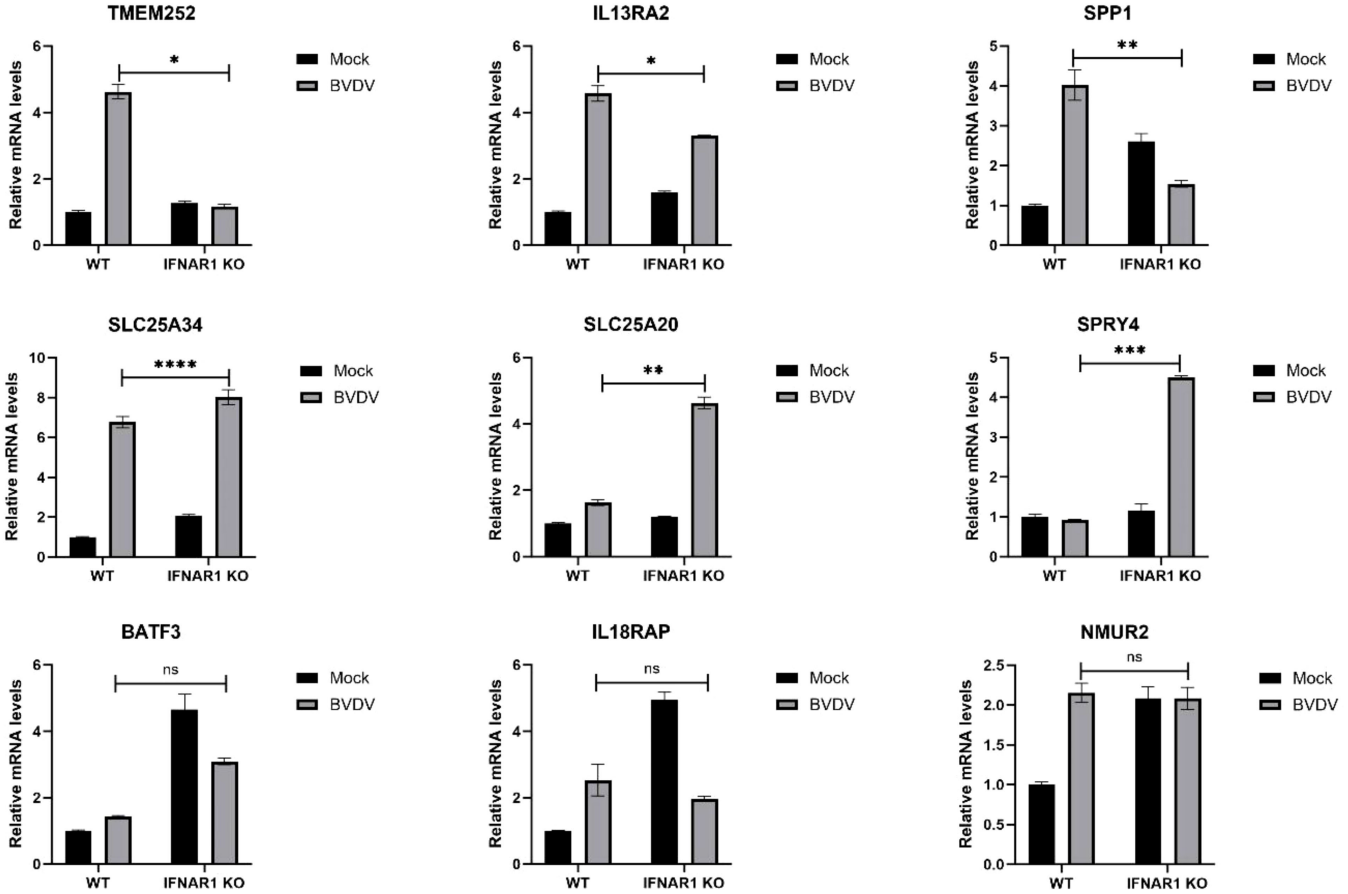
Validation of transcriptomic data with RT-qPCR. The genes were verified to be up-regulated in both WT and IFNAR1 KO MDBK cells using RT-qPCR assay. *p < 0.05; **p < 0.01; ***p < 0.001; ****p < 0.0001; ns, non-significant.

The expression of distinct genes was detected, and the reliability of the transcriptome data was confirmed using RT-qPCR. First, total RNA from WT and IFNAR1 KO MDBK cells in the infected and uninfected BVDV groups was extracted and analyzed by RT-qPCR. The 9 DEGs that were significantly up-regulated and common between the infected and uninfected WT and IFNAR1 KO MDBK groups were randomly selected for validation. The expression of the 9 DEGs increased consistent with the findings of RNA-seq data (**Figure 6**). Among the infected and uninfected WT and IFNAR1 KO MDBK groups, the degree of upregulation of TMEM252, IL13RA2, and SPP1 in the WT group was significantly higher than that in the IFNAR1 KO MDBK group, suggesting that these genes are regulated by IFNAR1, to exert its antiviral role. SLC25A34, SLC25A20, and SPRY4 were significantly more up-regulated in the IFNAR1 KO MDBK group than the WT group, suggesting that they regulate viral replication by mediating the cell growth cycle. The expression of BATF3, IL18RAP, and NMUR2 between the infected and uninfected WT and IFNAR1 KO MDBK groups was similarly up-regulated, indicating that the upregulation of these genes is not influenced by IFNAR1.

### 3.7 Promotion of BVDV replication upon knockdown of SLC25A34 and IL13RA2

**Figure 7:**
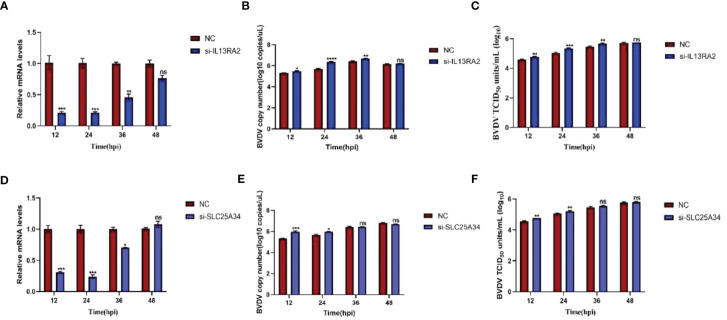
Effects of knockdown IL13RA2 and SLC25A34 genes on BVDV replication in IFNAR1 KO cells. **(A, D)** Relative mRNA levels of IL13RA2 and SLC25A34 in IL13RA2-knockdown of IFNAR1 KO cells and SLC25A34-knockdown of IFNAR1 KO cells infected with BVDV at 0.1 MOI, respectively. Transfected cells with si-IL13RA2, si-SLC25A34, and si-NC for 12 h and infected BVDV at 0.1 MOI, collected cells at designated time points postinfection and perform RT-qPCR with specific primers to determine relative mRNA levels. **(B, E)** Cells were transfected with si-IL13RA2, si-SLC25A34 and si-NC for 12 h respectively and infected with BVDV at 0.1 MOI, and viral fluids were harvested at different time points postinfection. Viral RNA levels of BVDV were determined and presented by RT-qPCR. **(C, F)** Viral titers were determined by TCID_50_ (Log10 TCID_50_/mL) from the viral samples as in **(B, E)**. *p < 0.05; **p < 0.01; ***p < 0.001; ns, non-significant.

Both transcriptomic data and RT-qPCR validation indicated that the expression of SLC25A34 and IL13RA2 significantly increased upon BVDV infection in WT and IFNAR1 KO MDBK cells. Therefore, the antiviral roles of these two genes were examined by silencing SLC25A34 and IL13RA2 using siRNA in IFNAR1 KO cells. The impact of IL13RA2 and SLC25A34 knockdown on BVDV replication was assessed through RT-qPCR and TCID_50_ assays. In IFNAR1 KO cells, knockdown of IL13RA2 significantly increased the RNA level and viral titer of BVDV within 36 h of knockdown, especially at 24 hpi (0.4 log10) (**Figures 7A–C**). At 12 hpi (0.3 log10) and 24 hpi (0.3 log10), SLC25A34 knockdown significantly increased the RNA levels and viral titers of BVDV (**Figures 7D–F**). The findings indicate that if the type I IFN pathway is compromised, IL13RA2 can be implicated in the initial and middle phases of cell control over BVDV infection replication, whereas SLC25A34 primarily contributes to the early stages of cellular regulation of BVDV infection.

### 3.8 IFNAR1/IFNAR2 double knockout promotes BVDV replication

**Figure 8:**
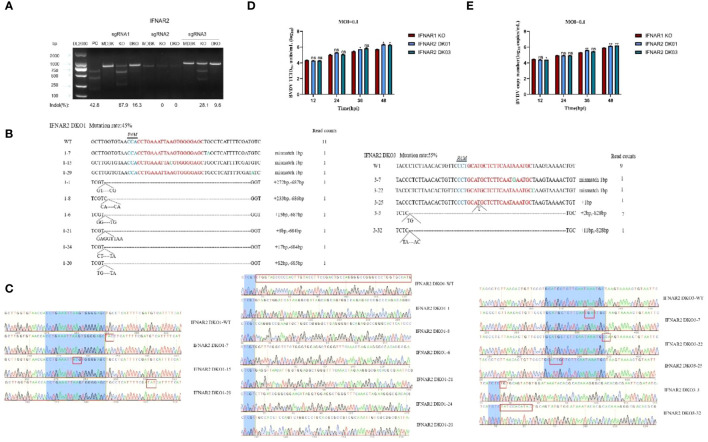
Construction of IFNAR2 DKO cell lines, and determination of the increase effect of IFNAR2 DKO cells on BVDV replication. **(A)** T7 endonuclease I cleavage detection to verify the sgRNA mutation efficiency. **(B)** The type of mutation in IFNAR2 DKO cells identified by TA cloning technique. **(C)** Sequencing results of mutation types of IFNAR2 DKO cells identified by TA cloning technique. **(D)** IFNAR1 KO MDBK (red) and IFNAR2 DKO (blue) cells were infected with BVDV at 0.1 MOI, respectively, and viral fluids were harvested at different time points postinfection. Viral titers as TCID_50_ (Log10 TCID_50_/mL) were determined. **(E)** Viral RNA levels of BVDV were determined and presented with RT-qPCR. In **(A, C, D)**, *p < 0.05, **p < 0.01, ns, non-significant.

To examine the synergistic effect of IFNAR1 and IFNAR2, we constructed double-knockout cell lines for IFNAR1 and IFNAR2. Three sgRNA knockout plasmids pKLV2-IFNAR2 sgRNAs for different exon positions were constructed and separately transfected into MDBK-Cas9 and IFNAR1 KO cells, respectively. Puromycin screening was performed to generate IFNAR2 knockout (IFNAR2 KO) and IFNAR1/IFNAR2 double-knockout (IFNAR2 DKO) cell lines. The T7EI digestion experiment verified the cleavage efficiency of the three sgRNAs and showed that sgRNA1 and sgRNA3 effectively mutated the IFNAR2 gene; therefore, sgRNA1 and sgRNA3 were used for further experiments (**Figure 8A**). The genomes of IFNAR2 DKO1 and IFNAR2 DKO3 cells were extracted for TA cloning. Sequencing analysis revealed various mutation types in the IFNAR2 gene (**Figures 8B, C**), with mutation efficiencies of 45% and 55%, respectively. To assess the impact of IFNAR2 knockout on viral replication, BVDV was introduced to IFNAR2 DKO1, IFNAR2 DKO3, and IFNAR1 KO cells at 0.1 MOI. Viruses were harvested at different time points of BVDV replication, and viral titers were determined in the medium by TCID_50_, and viral RNA level quantified by RT-qPCR. The results of viral titer assays showed that IFNAR2 DKO1 cells had a significant increase in BVDV titers at 36 hpi (0.4 log10) and 48 hpi (0.6 log10) compared to IFNAR1 KO cells, whereas IFNAR2 DKO3 cells had a significant effect at 48 hpi (0.6 log10) (**Figure 8D**). The collection of viral RNA at various time points was conducted, followed by extraction and reverse transcription into cDNA. RT-qPCR was used to detect the amount of BVDV viral RNA, and the findings were in agreement with the TCID_50_ results (**Figure 8E**). The findings indicate that IFNAR1/IFNAR2 double knockout promotes BVDV replication, and the receptor IFNAR1 plays an important role in IFNAR1/IFNAR2 dimer formation in innate immunity against virus invasion.

## 4 Discussion

### 4.1 Construction of the IFNAR1 KO MDBK cell line

Type I IFN pathway is an important protective mechanism for eukaryotic cells against viral invasion and impediment to efficient viral replication. MDBK cells are highly tolerant to a wide range of bovine-derived viruses and are an important tool for the isolation, culture, and study of bovine-derived viruses (2, 17–19). However, several viruses such as BVDV, proliferate slowly in MDBK cells with low viral titers, making isolation and characterization of clinical strains challenging.

In this study, the IFNAR1 KO MDBK cell line has been created by using the CRISPR/Cas9 genome editing technology. Then we used real-time PCR for relative quantification to validate IFNAR1 KO. The RNA-seq data revealed that in the IFNAR1 KO MDBK cell line, genes related to the type I IFN pathway, such as IFNAR1, STAT3, IRF9, and SOCS3, were significantly down-regulated. Compared to WT cells, IFNAR1 KO cells infected with BEV, BPIV-3, and BVDV showed inhibition of ISG expression, including IFIT5, IRF1, ISG15, and Mx (20, 21). In short, we knocked out the IFNAR1 gene in IFNAR1 KO MDBK cell line.

### 4.2 Potential mechanism of improved viral growth in IFNAR1 KO MDBK cells

We confirmed that the IFNAR1 KO MDBK cells could significantly increase the replication efficiency of BEV, BPIV-3, and BVDV from three lines of evidence: virus titration and detection at both RNA and protein levels. Then, comprehensive RNA-seq analysis was performed by using BVDV infection as an example to identify the potential mechanism. First, the genes related to the type I IFN pathway, such as IFNAR1, STAT3, IRF9, and SOCS3, and ISGs, including IFIT5, IRF1, ISG15, and Mx, were significantly downregulated, compared with BVDV-infected WT MDBK group.

Second, other innate immune pathways associated with DEGs in BVDV-infected and -uninfected IFNAR1 KO MDBK group, such as TGF-β and mTOR pathways, were significantly down-regulated compared with infected and uninfected WT group. For example, the TGF-β upstream signaling molecule BMP2, TGFβ2, and the downstream signaling molecules SMAD9; the mTOR upstream signaling molecule WNT5, WNT11, and the downstream signaling molecules LPIN were significantly down-regulated in the IFNAR1 KO MDBK group. Notably, Hippo, as an atypical innate immune pathway component, was significantly down-regulated in the IFNAR1 KO MDBK group, by crossing genes with Wnt and TGF-beta signaling pathways, such as the upstream signaling molecule TGF-β3, FZD1, and the downstream signaling molecule ID1. These results suggest that the downregulation of Hippo, TGF-β and mTOR pathways promotes viral replication in IFNAR1 KO MDBK cells. The interaction between the type I IFN pathway and Hippo, TGF-β, and mTOR pathways must be investigated.

Type I IFN receptor, a transmembrane receptor on the cell surface, consist of two subunits, IFNAR1 and IFNAR2 (22). When IFNAR1 and IFNAR2 are activated into dimers to form specific transmembrane protein complexes, they activate downstream receptor-associated proteins and exert antiviral effects. We confirmed this synergistic effect in IFNAR1/IFNAR2 DKO cells by showing that BVDV replication was promoted at 48 hpi.

### 4.3 The antivirus defense mechanism independent of the IFN- I pathway

The increase in virus growth in IFNAR1 KO cells was limited to < 1.5 log10, implying that some antiviral mechanisms are independent of the IFN- I pathway. For example, studies have shown that the MAPK pathway plays an important role in antiviral function (23, 24). In our study, DEGs such as EREG, DUSP5, and HSPA6, critical for the MAPK pathway, are significantly up-regulated in BVDV-infected IFNAR1 KO MDBK group, which is in agreement with other findings. EREG is upstream, whereas DUSP5 and HSPA6 are downstream, of MAPK pathways (25). Regarding the nonsignificant change of involved in the MAPK pathway in infected and uninfected WT cells, we hypothesize that the host cells activate the MAPK pathway to maintain antiviral function in the absence of type I IFN pathway. However, further investigation must confirm this hypothesis.

Among the specific DEGs in BVDV-infected IFNAR1 knockout cells, significantly up- and down-regulated genes reflect the adaptability and response of the cells in antiviral defense. Firstly, the most significantly up-regulated gene, MMP3, exhibits antiviral activity against various viruses such as dengue virus, vesicular stomatitis virus, and influenza A virus, through the activation of the NF-κB pathway (26, 27). This mechanism effectively defends against the threats posed by multiple viruses, exhibiting robust defensive capabilities. Additionally, SLC2A1 has been shown to increase cellular glucose uptake, providing energy for virus replication (28). Therefore, the upregulation of SLC2A1 after IFNAR1 knockout may synergistically promote BVDV replication (**Figure 4E**). Conversely, the significant downregulation of MGAT2 may reduce the n-acetylglucosamine modification on the cell surface, thereby affecting the interaction between the virus and host cells, and inhibiting virus replication (**Figure 4F**) (29). Although the specific roles of some DEGs in antiviral defense are unclear, the upregulation and downregulation of immune system-related DEGs, including SEMA3A (30), PADI1 (31), and MAP2K6 (32), as well as cell survival-related DEGs like STC1 (33), HSPA6 (34), and FGF17 (35), reflect the complex adaptability of BVDV-infected IFNAR1 knockout cells in antiviral defense. Further research will contribute to a deeper understanding of the specific roles and mechanisms of these genes in antiviral defense.

The expression of both SLC25A34 and IL13RA2 DEGs were similarly induced in BVDV-infected and -uninfected WT and IFNAR1 KO MDBK group, suggesting that both genes display antiviral roles independent on type I IFN pathway. SLC25A34 is a member of the SLC25 family, with an unclear function. It comprises 53 proteins and is located in the inner mitochondrial membrane, possibly functioning as a carrier for transporting biomolecules and affects lipid metabolism (36, 37). Studies have shown that miRNA-122 could disrupt lipid homeostasis by inhibiting SLC25A34 expression; meanwhile, SLC25A34 KO hepatocytes, lipid homeostasis was disrupted either (37). Because BVDV replication was reported to requires lipid, we inhibited SLC25A34 expression in IFNAR1 KO MDBK cells and determined that SLC25A34 knockdown could promote BVDV replication by 0.3 log10 at the early stage (12–24 hpi), consistent with other reports (38). In addition, IL13RA2, a receptor gene in the JAK-STAT pathway, was upregulated in host cells infected with infectious salmon anemia virus (39), and coinfected with hepatitis C virus and human immunodeficiency virus (40). In this study, knockdown of IL13RA2 in IFNAR1 KO cells promoted BVDV replication in the early and intermediate stages of BVDV infection (12–36 hpi), suggesting that IL13RA2 displays antiviral effect in IFNAR1 KO cells.

**Figure 9:**
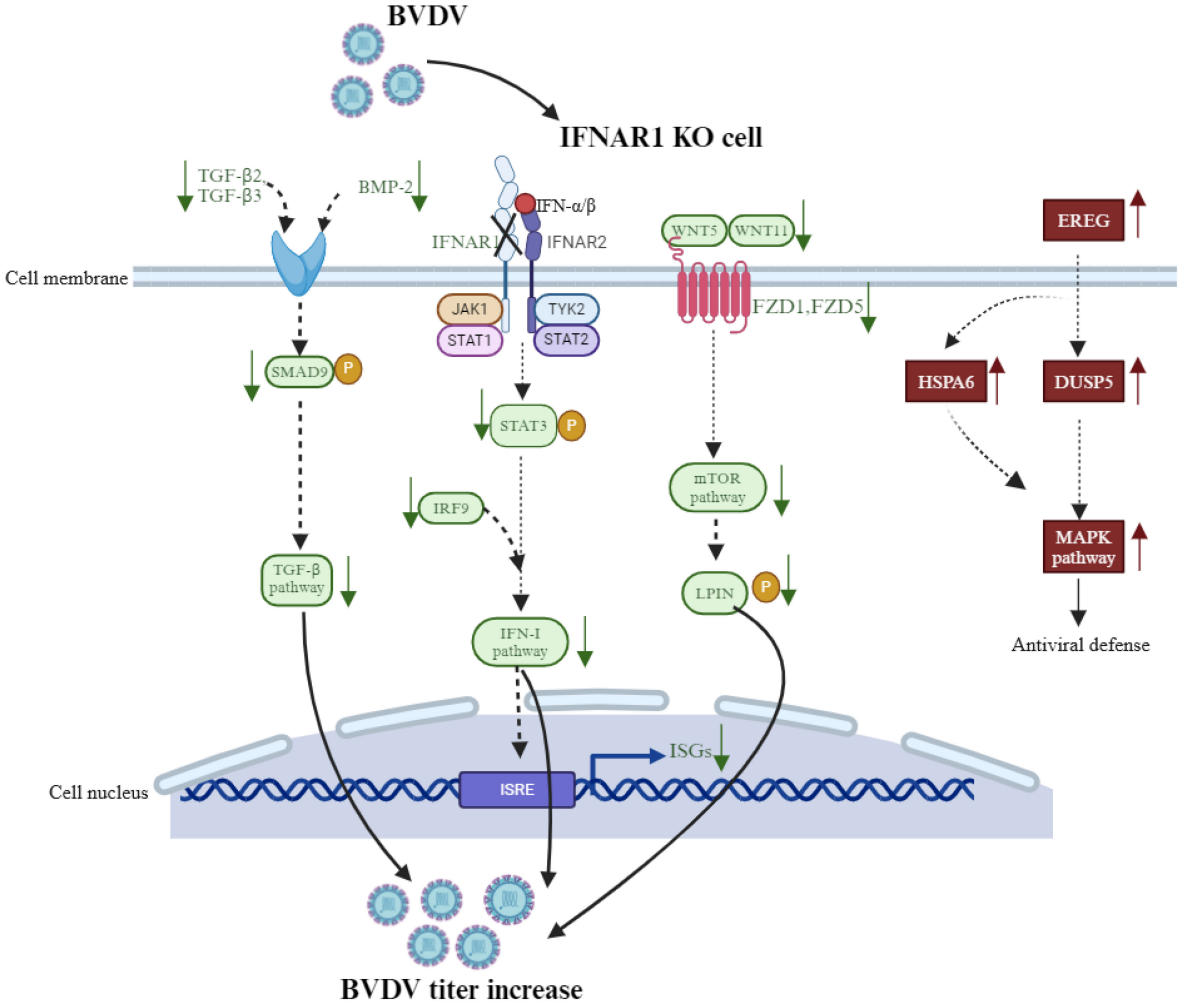
Schematic to review the potential mechanisms of IFNAR1 knockout to affect BVDV replication in MDBK cell line.

Taken altogether, IFNAR1 KO impairs the function of the IFN-I pathway, which significantly increases the efficiency of virus replication. Hippo, TGF-β, and mTOR pathways were inhibited, each which may play a synergistic role in promoting viral replication. Conversely, other mechanisms, such as activation of MAPK pathway and upregulation of SLC25A34 and IL13RA2, may exert antiviral effect to restrict excessive viral growth in the absence of the IFN-I pathway (**Figure 9**).

Based on this study, we not only gain a deeper understanding of the mechanisms of viral infection in cattle, but it also provides us with valuable insights for developing novel therapeutic and control strategies. Firstly, IFNAR1, a crucial receptor for type I interferon, plays a pivotal role in the innate immune system. The absence of this receptor significantly reduces the cellular response to IFNs, compromising the antiviral defense mechanism and allowing viral titers to surge. This underscores the centrality of IFNAR1 in antiviral immunity. Furthermore, the elevated viral titers in IFNAR1-knockout cells hold immense potential in controlling bovine viral infections. This knockout cell model serves as a robust platform to evaluate the effectiveness of antiviral drugs in the absence of an IFN response. Additionally, due to the heightened viral titers in these cells, they demonstrate potential applications in vaccine development. Moreover, we can leverage the IFNAR1 knockout cell model for virus screening and identification, thereby providing technical support for the prevention and control of bovine viral infections.

In conclusion, this study has constructed the IFNAR1 knockout MDBK cell line that can support better replication of bovine viruses, such as BVDV, BEV, and BPIV-3. Additionally, a potential mechanism on antiviral defense has been proposed.

## Data availability statement

The datasets presented in this study can be found in online repositories. The names of the repository/repositories and accession number(s) can be found below: PRJNA1060876 (SRA).

## Ethics statement

The animal study was approved by Ethics Committee for Animal Experimentation of Huazhong Agricultural University. The study was conducted in accordance with the local legislation and institutional requirements.

## Author contributions

YG: Conceptualization, Formal analysis, Investigation, Methodology, Resources, Software, Validation, Visualization, Writing – original draft. CJ: Conceptualization, Formal analysis, Investigation, Methodology, Resources, Validation, Writing – review & editing. HY: Conceptualization, Formal analysis, Investigation, Methodology, Resources, Validation, Writing – review & editing. QX: Formal analysis, Investigation, Methodology, Resources, Validation, Writing – review & editing. XX: Investigation, Methodology, Validation, Writing – review & editing. KY: Investigation, Methodology, Validation, Writing – review & editing. XY: Investigation, Methodology, Validation, Writing – review & editing. JC: Project administration, Writing – review & editing. YC: Project administration, Writing – review & editing. XC: Project administration, Writing – review & editing. LZ: Project administration, Writing – review & editing. CH: Conceptualization, Funding acquisition, Project administration, Supervision, Writing – review & editing. AG: Writing – review & editing, Conceptualization, Funding acquisition, Project administration, Supervision.
