# Supplementary material for: Construction of an IFNAR1 knockout MDBK cell line using CRISPR/Cas9 and its effect on bovine virus replication
Source: Front Immunol. 2024 Jul 19;15:1404649. doi: 10.3389/fimmu.2024.1404649 (PMC11294105; doi:10.3389/fimmu.2024.1404649)
Supplement: Supplementary file 1 [file DataSheet_1.pdf]

Supplementary Material

1 Supplementary Figures and Tables

1.1 Supplementary Figures

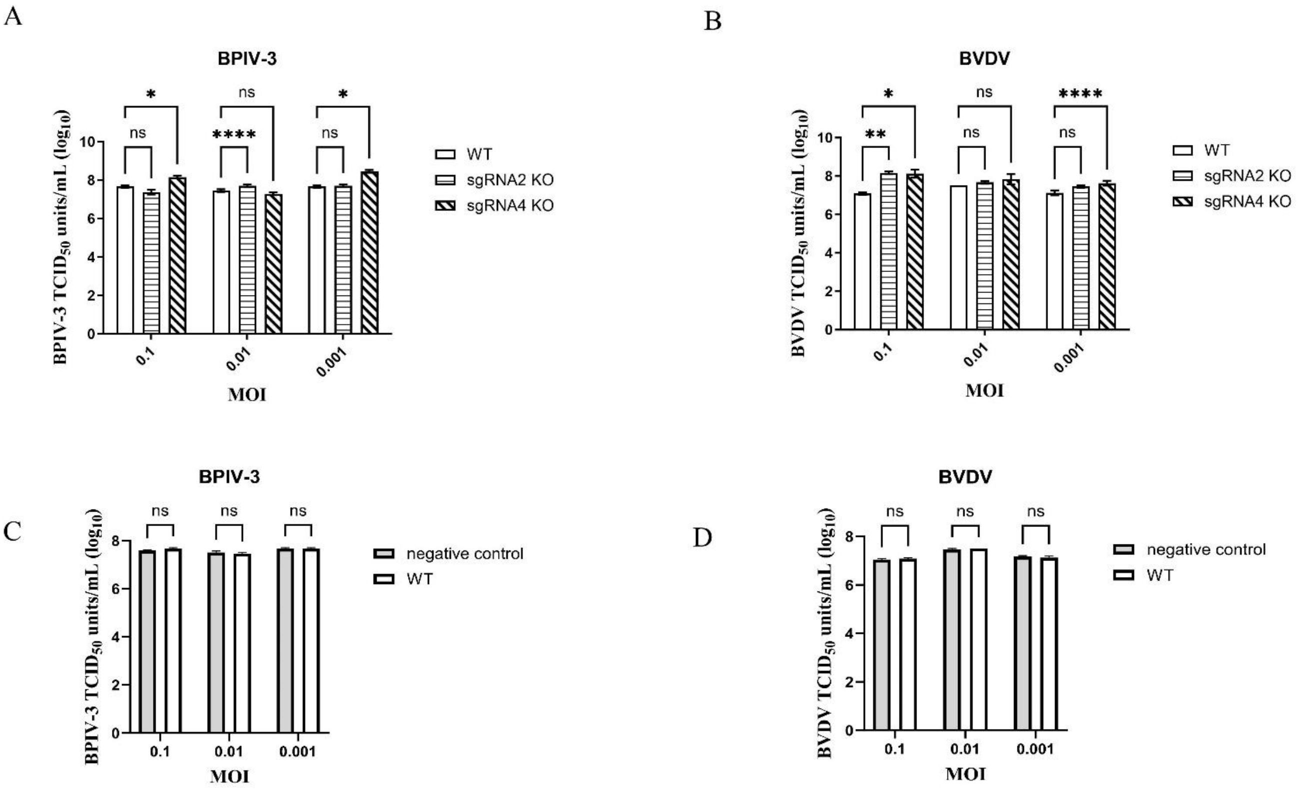

Supplementary Figure 1. Validation of sgRNA knock-out polyclonal cell phenotypes. (A) BPIV-3 infected WT, sgRNA2 KO and sgRNA4 KO MDBK cells with different MOI for 36 h and then collected samples to determine the viral titer. (B) BVDV infected WT, sgRNA2 KO and sgRNA4 KO MDBK cells with different MOI for 72 h and then collected samples to measure viral titers. (C) BPIV-3 infected WT and negative control cells with different MOI for 36 h and then collected samples to determine the viral titer. (D) BVDV infected WT and negative control cells with different MOI for 72 h and then collected samples to measure viral titers. Viral titers were determined by $TCID_{50}$ (Log10 $TCID_{50}$/mL). * $p < 0.05$; ** $p < 0.01$; ****$p < 0.0001$; ns, non-significant.

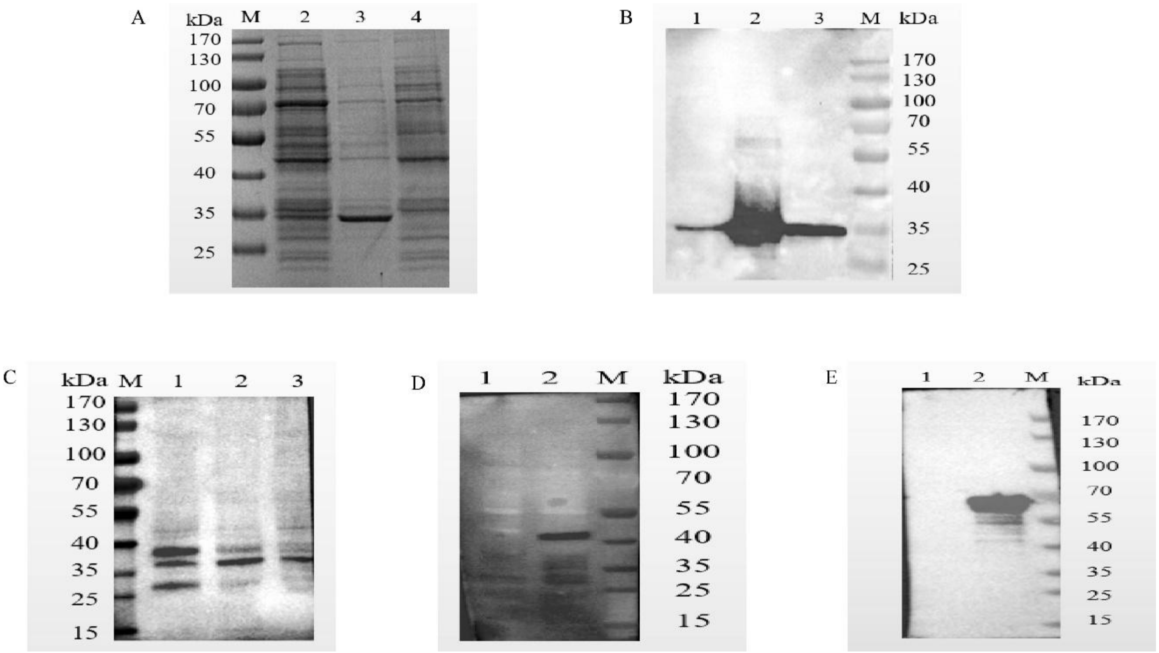

Supplementary Figure 2. SDS-PAGE and western blot analysis of antibodies to BEV, BVDV and BPIV-3. (A) expression of the recombinant BEV-VP2 with 10% SDS-PAGE. Lane 2, the supernatant of the bacterial culture tranformed with pET-BEV-VP2 after IPTG induction; Lane 3, the bacterial pellet tranformed with pET-BEV-VP2 after IPTG induction; Lane 4, the bacterial pellet transformed with pET-28-a empty vector as negative control after IPTG induction. (B) western blot analysis of the purified BEV-VP2. Lane 1, the supernatant of the bacterial culture tranformed with pET-BEV-VP2 after IPTG induction; Lane 2, the bacterial pellet tranformed with pET-BEV-VP2 after IPTG induction; Lane 3, the purified BEV-rVP2. (C) Validation with western blotting assay of BEV reaction with BEV-VP2 polyclonal antibodies in rabbit antiserum. Lane 1, The infected MDBK cells at 12 h after BEV infection; Lane 2, The infected MDBK cells at 30 min after BEV infection; Lane 3, The MDBK cells without BEV-infection as negative control. (D) Validation of BVDV reaction with BVDV monoclonal antibody. Lane 1, MDBK cells at 24 h after BVDV infection; Lane 3, MDBK cells without BVDV-infection. (E) Validation of BPIV-3 reaction with BPIV-3 monoclonal antibody. Lane 1, MDBK cells at 24 h after BPIV-3 infection; Lane 3, MDBK cells without BPIV-3-infection. Lane M in A-E represents markers of the reference proteins for molecular mass.

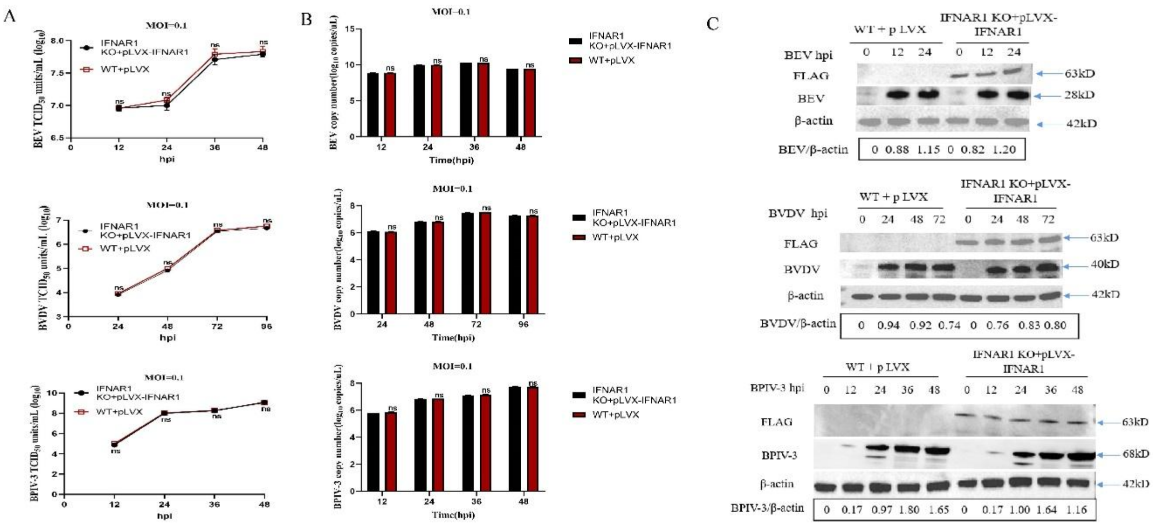

Supplementary Figure 3. Complementary of IFNAR1 in IFNAR1 KO MDBK cells restored IFNAR1 function. (A) WT and IFNAR1 KO MDBK cells were transfected with flag tagged empty vector and IFNAR1, respectively and infected separately with BEV, BVDV and BPIV-3 at 0.1 MOI. Then the infected cells were harvested at different time points and their viral titers were determined by $TCID_{50}$ (Log10 $TCID_{50}$/mL). (B) The genomic RNA levels of BEV, BVDV and BPIV-3 in the same samples as (A) were determined by RT-qPCR and presented. (C) Total cell lysates were prepared in parallel to A and B. Western blot assay was used to detect the replication of BEV, BVDV and BPIV-3 by using β-actin as an internal reference. ns, non-significant.

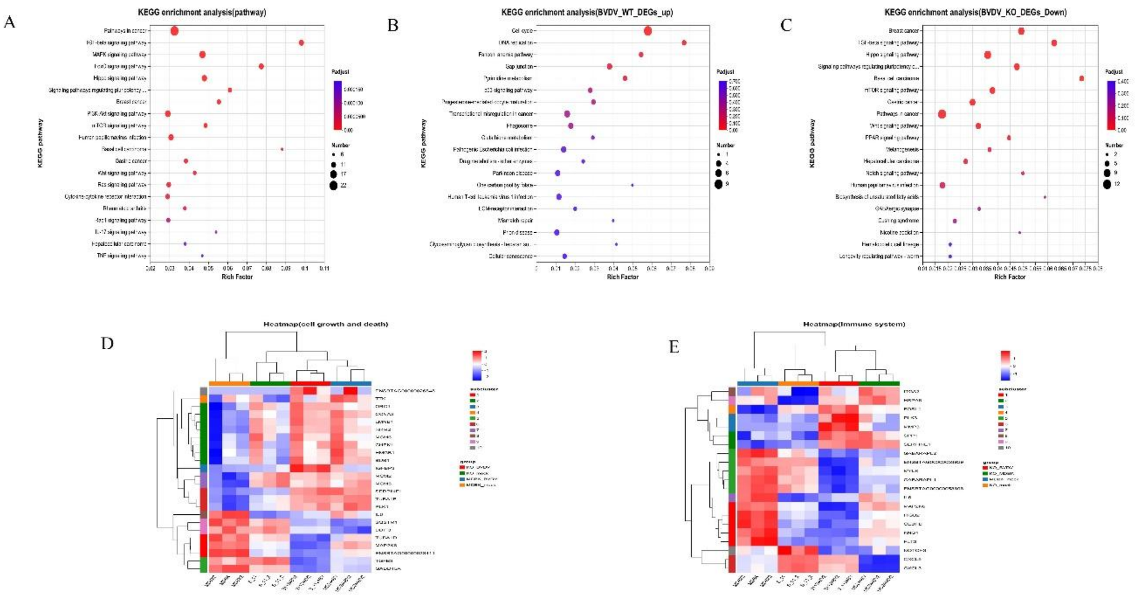

Supplementary Figure 4. KEGG enrichment analysis and heatmap of DEGs. (A) Significant enriched pathways for the DEGs. (B) KEGG enrichment analysis of upregulated DEGs in BVDV-infected and -uninfected WT MDBK groups. (C) KEGG enrichment analysis of downregulated DEGs in BVDV-infected and -uninfected IFNAR1 KO MDBK groups. (D) and (E) Heatmap analysis

of DEGs in cell growth and death and the immune system, respectively. Upregulated DEGs are indicated in red, while downregulated DEGs in blue.

1.2 Supplementary Tables

Supplementary Table 1. The primers of IFNAR1 and IFNAR2 used for sequencing and T7EI detection.

Gene	Sequence（5’-3’）
IFNAR1-1-T7EI- F	CACAGCTCAGATTGGTCCCC
IFNAR1-1-T7EI-R	GCAAAACTTCCTTCTTACCTGTGG
IFNAR1-2,3-T7EI-F	CTGGCCTATTACAGGTGCTCA
IFNAR1-2,3-T7EI-R	ACACAGTCTTTTTACCTCAGCAT
IFNAR1-4-T7EI-F	GTGCCTCAGTCTCCGTCGC
IFNAR1-4-T7EI-R	TGAGTCCGTTTCAATGAGGCAC
IFNAR2-1-T7EI-F	CTGCCACAGAGTCCCTCTCGT
IFNAR2-1-T7EI-R	CTAGTTTGAAACCCAGCCTCCACC
IFNAR2-2-T7EI-F	AGGCAAATTGCCTTGAACAGGTAAACA
IFNAR2-2-T7EI-R	ACACCAAGCATGGACTAACGG
IFNAR2-3-T7EI-F	ATGACAGTCTGCCCACTCATCTC
IFNAR2-3-T7EI-R	TGTGCGTGTATTTATCCACATACTGCA

Supplementary Table 2. The primers of BEV-VP2 used for recombinant plasmid construction.

Gene	
BEV-VP2-1F	AATGGGTCGCGGATCCTCACCAAGTGCTGAGGCCT
BEV-VP2-1R	GGTGGTGGTGCTCGAGGGTGGATCCGGTGGCATATG

Supplementary Table 3. The primers of IFNAR2 used for plasmid construction.

NO. sgRNA	sgRNA sequence	Plasmid construction primer sequence（5'-3'）
sgRNA1	GCTCCCCACTTAATTTCAGG	F: CACCGGCTCCCCACTTAATTTCAGG
		R: AAACCCTGAAATTAAGTGGGGAGCC
sgRNA2	GGAGGACAGTGACTCCACGG	F: CACCGGGAGGACAGTGACTCCACGG
		R: AAACCCGTGGAGTCACTGTCCTCCC
sgRNA3	GCATTTATTGAAGAGCATGC	F: CACCGGCATTTATTGAAGAGCATGC
		R: AAACGCATGCTCTTCAATAAATGCC
